# Identification of mammalian orthologs using local synteny

**DOI:** 10.1186/1471-2164-10-630

**Published:** 2009-12-23

**Authors:** Jin Jun, Ion I Mandoiu, Craig E Nelson

**Affiliations:** 1 Computer Science & Engineering Department, University of Connecticut, Storrs, CT 06269, USA; 2 Department of Molecular and Cell Biology, University of Connecticut, Storrs, CT 06269, USA

## Abstract

**Background:**

Accurate determination of orthology is central to comparative genomics. For vertebrates in particular, very large gene families, high rates of gene duplication and loss, multiple mechanisms of gene duplication, and high rates of retrotransposition all combine to make inference of orthology between genes difficult. Many methods have been developed to identify orthologous genes, mostly based upon analysis of the inferred protein sequence of the genes. More recently, methods have been proposed that use genomic context in addition to protein sequence to improve orthology assignment in vertebrates. Such methods have been most successfully implemented in fungal genomes and have long been used in prokaryotic genomes, where gene order is far less variable than in vertebrates. However, to our knowledge, no explicit comparison of synteny and sequence based definitions of orthology has been reported in vertebrates, or, more specifically, in mammals.

**Results:**

We test a simple method for the measurement and utilization of gene order (local synteny) in the identification of mammalian orthologs by investigating the agreement between coding sequence based orthology (Inparanoid) and local synteny based orthology. In the 5 mammalian genomes studied, 93% of the sampled inter-species pairs were found to be concordant between the two orthology methods, illustrating that local synteny is a robust substitute to coding sequence for identifying orthologs. However, 7% of pairs were found to be discordant between local synteny and Inparanoid. These cases of discordance result from evolutionary events including retrotransposition and genome rearrangements.

**Conclusions:**

By analyzing cases of discordance between local synteny and Inparanoid we show that local synteny can distinguish between true orthologs and recent retrogenes, can resolve ambiguous many-to-many orthology relationships into one-to-one ortholog pairs, and might be used to identify cases of non-orthologous gene displacement by retroduplicated paralogs.

## Background

The accurate determination of orthology is central to comparative genomics. Pinpointing the origin of new genes, understanding the evolution of new gene families, and assessing the impact of gene and genome duplication events all require the accurate assignment of orthology between genes in distinct genomes. In complex genomes with large gene families this task requires differentiating between genes that have diverged through a speciation event (orthologs) and those derived through duplication events within a species (paralogs). Determination of orthology and paralogy is especially challenging in vertebrates species such as the mammals. Very large gene families, high rates of gene duplication and loss, multiple mechanisms of gene duplication, and high rates of retrotransposition all combine to make the determination of orthology between vertebrate genes difficult.

Given the importance of accurate orthology assignment, many methods have been developed to identify orthologous genes. Most of these methods rely upon analysis of the inferred protein sequence of the genes in question by clustering the results of protein sequence comparisons results in the classification of putative orthologs. Examples of this approach include reciprocal best BLAST hits and more inclusive BLAST based clustering methods. Splitting of these clusters based on relative similarity can distinguish between older and newer duplication events and is implemented in the widely used Inparanoid algorithm [1] and related approaches [2]. While these methods are robust and easily implemented, they rely upon a single character, the protein sequence, for classifying genes into orthologous groups.

More recently, methods have been proposed that use genomic context in addition to protein sequence to improve orthology assignment. These methods have been most successfully implemented in fungal genomes [3,4], and have long been used in prokaryotic genomes [5,6], where gene order is far less variable than in eukaryotes. An interesting implementation of this approach is found in the SOAR and MSOAR algorithms [7,8], which seek to assign orthology by minimizing the recombination distance between two genomes. In most of these approaches, synteny blocks covering some percentage of the genome are used hierarchically with protein coding information to assign orthology between similar genes. Approaches that exploit synteny information can be particularly useful in resolving ambiguous sequence based matches between putative orthologs. Recently, Han and Hahn [9] used local synteny information to identify parent-daughter relationships among duplicated genes. However, it is worth noting that "phylogenetic shadowing" [10] approaches used in genome assembly might lead to a lack of independence between sequence and synteny information.

In this study, we evaluate a simple method for using gene order (local synteny) in the identification of mammalian orthologs. We explicitly compare the relative performance of local synteny and Inparanoid for inferring orthology within this clade and show that local synteny alone is sufficient to identify orthologs with an accuracy comparable to that of Inparanoid. By analyzing cases of discordance between local synteny and Inparanoid we show that local synteny can distinguish between true orthologs and recent retrogenes, can resolve ambiguous many-to-many orthology relationships into one-to-one ortholog pairs, and can highlight possible cases of parental gene displacement by retrogenes.

## Results

### Using Local Synteny to Infer Orthology

**Figure 1:**
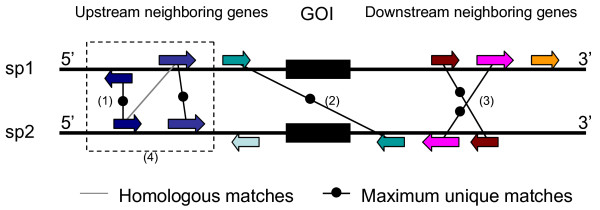
**Diagram illustrating the computation of the maximum number of unique homologous matches**. We counted the homologous matches between 3 neighboring genes (shown as filled arrows with corresponding gene orientations) on each side of the two genes of interest (GOI, shown as two black boxes). Homology between neighboring genes (shown as line between genes) is defined as Blastp E-value < 1e-5. The homologous matches do not need to be between the genes with the same orientations (1) or on the same strand (2). Also they do not need to be co-linear (3). When there are many-to-many homologous matches, we choose the maximum unique matches (4). The number of maximum unique homologous matches in this case is 5.

Several orthology inference methods, such as Inparanoid [1] and OrthoMCL [2], use coding sequence similarity (for example Blastp score [11] or Protdist [12]) as primary orthology signal. In this paper, we use local synteny information to determine orthology. We define the local synteny of two genes as the maximum number of unique homologous matches between their six neighboring genes (three upstream and three downstream immediate neighbors for each gene, see Figure 1). Homology between two neighboring genes is defined as Blastp E-value < 1e-5.

**Figure 2:**
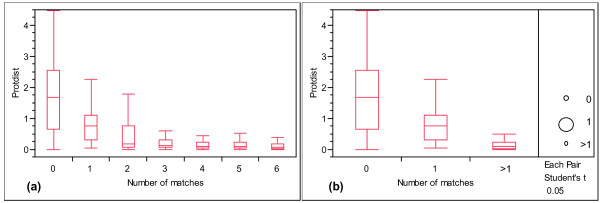
**The box plot of Protdist in each level of local synteny**. Local synteny is measured by the maximum number of unique homologous matches between 6 neighboring genes. (a) It shows a negative relationship between Protdist and the number of matches (r = -0.67 with p-value < 0.0001). (b) No match, one match and more than one match have significantly different Protdist means (Student's t test, p-value < 0.05). Protdist and the numbers of matches are calculated from the randomly sampled 1,000 cross-species homologous protein pairs (defined as belonging to the same Pfam families).

To validate the use of local synteny for inferring orthology, we evaluated the correlation between Protdist and local synteny using a dataset derived from the Pfam protein family database. Pfam families are highly accurate protein families based on protein domains [13]. We randomly selected 1,000 cross-species homologous protein pairs (homologs belonging to a given Pfam family) from five mammalian genomes: *Homo sapiens *(human), *Pan troglodytes *(chimp), *Mus musculus *(mouse), *Rattus norvegicus *(rat), and *Canis familiaris *(dog). To avoid protein family-specific bias in this analysis, we chose one homologous pair from each Pfam family. For each pair we computed Protdist and the degree of local synteny between the two genes. Figure 2a shows that there is negative correlation between Protdist and the local synteny of these samples (r = -0.67 with p-value < 0.0001). This is not surprising as gene order is conserved between DNA segments resulting from speciation or large-scale segmental duplication events. However, because local synteny is not directly computed based on the coding sequence, we can use local synteny to test hypotheses of orthology between two genes independent of their coding sequence.

Theoretically two non-orthologous genes should not share homologous neighboring genes. However, there is a small probability of homology matches occurring by chance. Moreover rearrangements, insertions, and deletions will lead to loss of local synteny between orthologous genes. In order to account for these events, we wanted to determine an optimal window size and match percentage that could reliably identify orthologs based on local synteny. In Figure 2b, we test the impact of the number of matches between six neighboring genes to determine local synteny. Student's t tests comparing Protdist means illustrate that 0, 1, and >1 matches have significantly different Protdist means (p-value ≤ 0.05). In order to choose the threshold of homologous matches, we calculate the false positive rate (FP) and false negative rate (FN) to Inparanoid orthologs and Ensembl orthologs then choose the threshold that minimizes the sum of FP and FN events. For six neighbors, the pairs with more than one homologous match minimizes FP and FN rates (0.152 to Inparanoid orthologs, and 0.151 to Ensembl orthologs (see Additional file 1)). Increasing the window size to 10 or 20 flanking genes does not show a significant difference in detecting orthology (see Additional file 1). Based on these results we define orthology by local synteny when the number of maximum unique homologous matches between the six neighboring genes is greater than one. We will refer to those pairs as syntenic from now on.

### False Positive/False Negative Rates Estimated by LCA (Latent Class Analysis)

Since many orthology detection methods use more complicated algorithms than just coding sequence similarity, a high correlation between Protdist and local synteny (Figure 2) is not sufficient evidence that local synteny captures true orthology. For a more rigorous analysis we compared local synteny based orthology to the orthology relationships inferred by six well-known orthology detection methods: Inparanoid [1], OrthoMCL [2], RBH (Reciprocal Best Hit), SBH (Single-way or One-way Best Hit), BLASTP, and orthology data from Ensembl [14]. Since there is no gold standard of orthology, we performed Latent Class Analysis (LCA) [15,16] to estimate the accuracy (sensitivity and specificity) in the absence of a reliable standard. LCA estimates false positive (FP) and false negative rates (FN) based on agreement and disagreement between various ortholog definitions. To minimize sampling bias 10 LCA's were performed on random samples with a size of 1,000 orthologous genes from five mammalian genomes using the same sampling method described in the previous section. For one sample 1,000 Pfam families were randomly selected, then one cross-species protein pair was selected from each Pfam family for analysis by the seven compared orthology inference methods. For all methods based on coding sequence similarity, the longest proteins were used as the representative proteins of the genes. A more detailed description can be found in the methods section.

**Figure 3:**
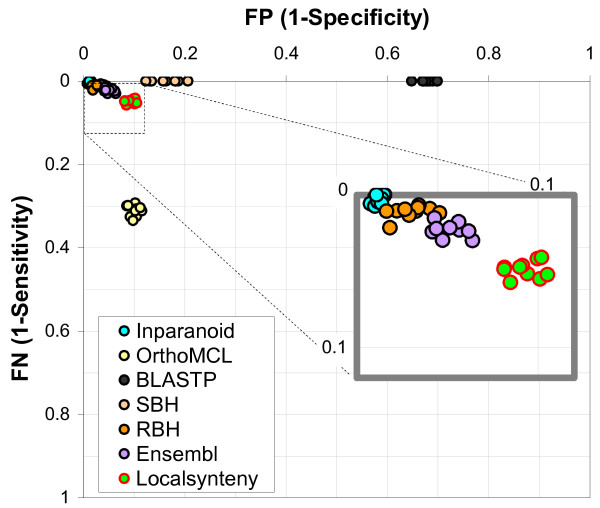
**Estimated false positive (FP) and false negative rates (FN) for seven orthology detection methods**. FP and FN rates are estimated for each method by using LCA from 10 sampling replicates. Inset figure shows the FP and FN rates of four orthology detection methods (Inparanoid, RBH, Ensembl and local synteny based orthologs) having lowest FP and FN rates. For details and each orthology detection method, see Methods.

Figure 3 shows that orthology inference based on local synteny yields a lower FP rate than SBH/BLASTP and a lower FN rate than OrthoMCL, reinforcing the interpretation that orthology can be accurately inferred without coding sequence information. However, local synteny has a slightly higher FN rate than the four orthology methods based on coding sequence similarity (BLASTP, SBH, RBH and Inparanoid). This is partially due to the fact that these coding sequence based methods cannot distinguish retrotransposed genes from the original copies unless retrotransposed genes are sufficiently diverse. This might lead to incorrect orthology assignments (retrotransposed copies replace the original ortholog genes) or ambiguous orthology assignments (one-to-many or many-to-many ortholog groups including retrotransposed copies as their members) by these methods. Local synteny also has a slightly higher FP rate than Inparanoid and RBH. This is likely due to the fact that local synteny cannot distinguish DNA-mediated duplicates from the original copies. We analyze these discordances in more detail in the following section.

### Discordance between Inparanoid Orthology and Local Synteny Based Orthology

**Figure 4:**
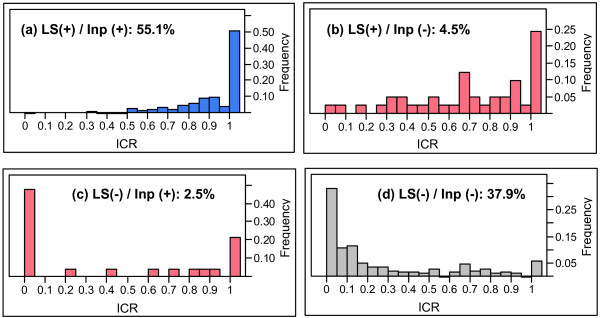
**Intron conservation ratio (ICR) histograms in four concordant and discordant cases between Inparanoid orthology and local synteny based orthology**. 7.0% of disagreement between Inparanoid orthology and local synteny based orthology and the majority (93%) of sample pairs are concordant between two orthology methods. Most of the pairs concordant between local synteny and Inparanoid, (a) syntenic Inparanoid ortholog (denoted as LS(+)/Inp(+) ) and (d) non-syntenic non-Inparanoid pairs ( LS(-)/Inp(-) ), are also concordant with ICR: orthologs have a high ICR and non-orthologs have a low ICR. However in discordant cases, (b) syntenic non-Inparanoid ( LS(+)/Inp(-) ) and (c) non-syntenic Inparanoid orthologs ( LS(-)/Inp(+) ), ICR histograms show a partial agreement with two orthology definitions. Also there are small numbers of non-syntenic non-Inparanoid pairs ( LS(-)/Inp(-) ) having perfect ICR in panel (d).

Because Inparanoid is one of the most widely used ortholog definition methods [17-19] and is purely based on the coding sequence information, we decided to do a more thorough comparison of Inparanoid and local synteny based orthology. Figure 4 shows the agreement and disagreement between these two orthology prediction methods. The majority of these samples are concordant between two ortholog predictions (syntenic/Inparanoid (55.1%) and non-syntenic/non-Inparanoid (37.9%)), which agree with the LCA results (Figure 3). However, 2.5% of Inparanoid orthologs are non-syntenic, and 4.5% of gene pairs are syntenic but not Inparanoid orthologs.

In order to identify the source of this discordance we employed intron-based evidence. Specifically, for each gene pair used in the test we computed the intron conservation ratio (ICR) between the two genes defined as follows (for more details see Methods):

Figure 4 shows ICR histograms for pairs of genes falling in each of the four classes of agreement/disagreement between Inparanoid and local synteny. In both concordance cases, namely for syntenic Inparanoid orthologs (Figure 4a) and non-syntenic non-Inparanoid orthologs (Figure 4d), ICR is in strong agreement with the orthology assignments made by the two methods. Indeed, most of the syntenic Inparanoid orthologs have ICR of 1 (Figure 4a), and the majority of non-syntenic non-Inparanoid orthologs have ICR < 0.5 (Figure 4d). In the two discordant cases (7% of the evaluated gene pairs) intron evidence can be used to resolve the conflicting assignments made by Inparanoid and local synteny. About 3/4 of syntenic non-Inparanoid orthologs (Figure 4b) have ICR > 0.5 and half of non-syntenic Inparanoid orthologs (Figure 4c) have ICR = 0. This suggests that in these cases local synteny based orthology assignments are more often concordant with gene structure evidence (ICR) than those based on coding sequence similarity. However, the ICR histogram of non-syntenic Inparanoid orthologs (Figure 4c) has a bimodal distribution, which might arise from a mixture of FNs from local synteny (pairs with high ICR) and FPs from Inparanoid (pairs with low ICR). We further investigate these cases in the following subsections.

### Non-syntenic Inparanoid orthologs with zero ICR: Retrotransposed copies

**Figure 5:**
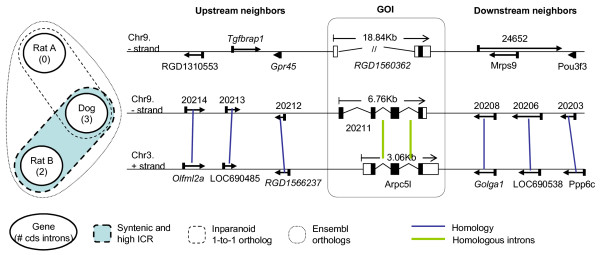
**One example of the RT miscall cases by Inparanoid which is confirmed with local synteny and ICR**. The Ensembl IDs of GOIs are ENSRNOG00000016444 (Rat A), ENSRNOG00000014317 (Rat B) and ENSCAFG00000020211 (Dog). Genes are shown corresponding to the strand of GOI in order to show the homology between neighboring genes. Five digits gene IDs are the last five digits of Ensembl gene IDs. IDs in italic typeset are predicted ones. Homology between two neighboring genes are defined by Blastp E-value < 1e-5.

All the non-syntenic Inparanoid ortholog pairs with zero ICR contain one intronless copy and one intron-bearing member. Based on local synteny information and intron conservation ratio, these intronless copies are probably retrotransposed (RT) copies of the original orthologs. Of all the samples, 1.2% are non-syntenic 0-ICR Inparanoid orthologs. Inparanoid likely included RT copies in these orthologous groups because the RT copies have not diverged sufficiently to be distinguished from their parent gene. In 0.2% of samples Inparanoid chose RT copies even though there were other syntenic high-ICR copies. Figure 5 shows one of these pairs. In this case, the Blastp score of the dog gene to the RT rat gene (shown as Rat A) is smaller than to the syntenic high-ICR rat gene (Rat B), which caused an Inparanoid miscall, assigning the RT paralog as the ortholog. For the detailed information including Blastp scores and Protdist in tabular format, see Additional file 2.

The rest of non-syntenic 0-ICR Inparanoid orthologs (1.0%) of all samples are classified as one-to-many orthologs by Inparanoid. In many of these one-to-many orthologs, syntenic high-ICR pairs are chosen as "core orthologs" (based on the Inparanoid scores) with additional RT copies added to the orthology group because their protein sequences are still close to those of the original copies. By using local synteny, the RT copies in one-to-many orthologs would be distinguished from the other members. Thus, local synteny based orthology separates RT copies from those generated by speciation or other duplication mechanisms and can be more informative in recovering the evolutionary history of a gene family.

### Non-syntenic Inparanoid orthologs with non-zero ICR: Loss of local synteny

All non-syntenic Inparanoid orthologs with an ICR > 0 (1.3%) are likely to result from the loss of local synteny. They each have one homologous match between neighboring genes (lower than the threshold of being syntenic) and are selected from distant species pairs (i.e. not human-chimp or mouse-rat). Each of these distant pairs is part of larger orthology groups (5-species), in which the counterparts in closer species pairs have higher local synteny (more than 1 match). This loss of local synteny likely results from rearrangements and gene insertions or gene losses in more distant species. See Additional file 3 for an example.

### Syntenic non-Inparanoid orthologs: Distant paralogs

There are 2.3% syntenic non-Inparanoid orthologs with an ICR ≥ 0.5 (Figure 4b). The majority (2.1/2.3%) of syntenic non-Inparanoid orthologs with high ICR are likely distant paralogs: both genes are in different syntenic Inparanoid orthology groups with high ICR. This may result from old DNA-mediated duplication events followed by speciation events without significant local genome rearrangements. Local synteny cannot distinguish between orthologs created by large-scale segmental duplications and by polyploidy events. Moreover, the syntenic non-Inparanoid pairs with an ICR < 0.5 (1.1% of all tested pairs) are also likely distant paralogs, by old tandem duplications with different gene structures resulting in lower ICR.

### Non-syntenic non-Inparanoid orthologs with ICR of 1: More distant paralogs

In the non-syntenic non-Inparanoid case (Figure 4d), where the most of these pairs have low ICR, we still find 2.2% of pairs with ICR equal to 1. All these pairs are likely distant paralogs. Table 1 contains the summary of all of these cases.

**Table 1:** Summary of disagreement among three measures: Inparanoid orthology, local synteny based orthology, and intron conservation ratio (ICR).

Orthology	ICR	Explanations
Non-syntenic Inparanoid orthologs	ICR = 0	RT miscalls (0.2%) Part of 1-many (1.0%)
	
	ICR > 0	Loss of local synteny (1.3%)

Syntenic non-Inparanoid orthologs	ICR ≥ 0.5	RT miscalls (0.2%) Distant paralogs (2.1%)
	
	ICR < 0.5	Distant paralogs (1.1%)

Non-syntenic non-Inparanoid orthologs	ICR = 1	Distant paralogs (2.2%)

### Local Synteny Breaks the Tie

Since local synteny is able to differentiate some speciation and duplication events, a phylogenetic tree (or duplication-speciation history) of ambiguous many-to-many orthologs may be determined using local synteny information. In mouse-to-rat ortholog definitions from Inparanoid, there are 131 many-to-many ortholog groups. The majority (~75%) of many-to-many groups are comprised of DNA-mediated duplicated copies (usually from tandem duplications combined with one or two distant segmental duplication events) while ~20% have true orthologs (confirmed by local synteny and ICR) as well as non-syntenic intronless copies (probably from RT events).

**Figure 6:**
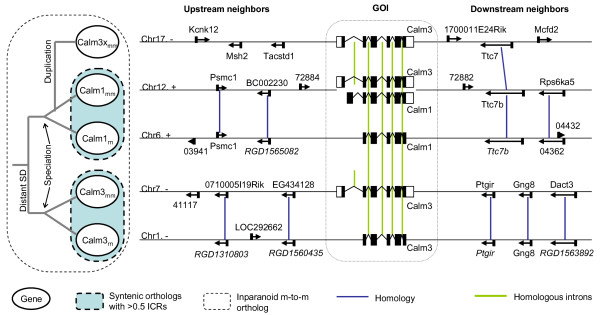
**One example of many-to-many Inparanoid ortholog groups where a SD event proceeded mouse-rat speciation**. The Ensembl gene IDs of GOI are ENSMUSG00000001175 (Calm1mm), ENSMUSG00000019370 (Calm3mm), ENSMUSG00000036438 (Calm3xmm), ENSRNOG00000004060 (Calm1rn), ENSRNOG00000016770 (Calm3rn). The gene structures and neighboring gene orders of five Calm1 and Calm3 genes in mouse and rat genomes are shown. The event tree on the left side is predicted based on the local synteny. SD: segmental duplication. Genes are shown corresponding to the strand of GOI in order to show the homology between neighboring genes. Five digits gene IDs are the last five digits of Ensembl gene IDs. IDs in italic typeset are predicted ones. Homology between two neighboring genes are defined by Blastp E-value < 1e-5.

We present two many-to-many Inparanoid ortholog groups where the local synteny determines the order of evolutionary events in the gene family. One of them is an example of orthologs from distant DNA-mediated duplication event(s) followed by possible rearrangements or gene gains/losses, and then speciation event (Figure 6). In this ortholog group, there are three mouse gene members, ENSMUSG00000001175 (MGI symbol: Calm1), ENSMUSG00000019370 (Calm3), ENSMUSG00000036438 (Calm3, which referred to Calm3x in the figure to avoid confusion) and two rat gene members, ENSRNOG00000004060 (Calm1), ENSRNOG00000016770 (Calm3). All the genes are on different chromosomes and the transcripts of these (protein coding) genes are known. Since all the cross-species pairwise sequence similarity measures are equal, Inparanoid could not pick distinct orthologs nor could phylogenetic tree-building programs determine the tree (for Blastp results see Additional file 4). Neither could ICR break the tie due to a high conservation of gene structure. Finally, no Ensembl ortholog prediction is made between these genes. However, in the figure, two Calm1 genes (ENSMUSG00000001175 and ENSRNOG00000004060) and two Calm3 genes (ENSMUSG00000019370 and ENSRNOG00000016770) have high local synteny (4 and 5 matches, respectively) and any other local synteny is either 0 or 1. In this specific case, local synteny helps break the tie in sequence based similarity. Using this information we can infer an old segmental duplication (SD) event before mouse-rat speciation giving rise to the Calm1 and Calm3 ancestors followed by rearrangements reducing the local synteny between these two mouse and rat ortholog pairs (Figure 6). The third mouse gene (Calm3x) has just one match with mouse Calm1 gene and rat Calm1 gene, and high levels of intron conservation, indicating a DNA duplication event, but we do not have enough local synteny information to tell precisely when the duplication occurred. Also since the mouse Calm3x gene has only one match with the rat Calm1 gene, local synteny does not find this apparent ortholog. For the detailed information in tabular format, see Additional file 4.

**Figure 7:**
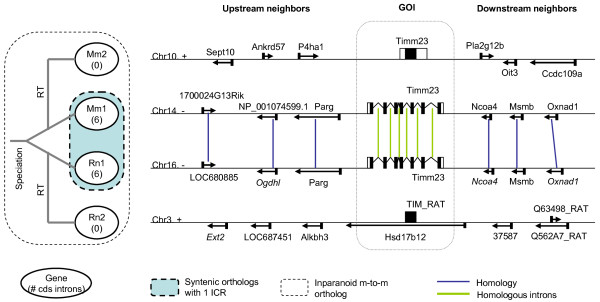
**One example of many-to-many Inparanoid ortholog groups where RT events followed the mouse-rat speciation**. The Ensembl gene IDs of GOI are ENSMUSG00000013701 (Mm1), ENSMUSG00000069622 (Mm2) and ENSRNOG00000019811 (Rn1), ENSRNOG00000032900 (Rn2). The gene structures and neighboring gene orders of four Timm23 genes in mouse and rat genomes are shown. The event tree on the left side is predicted based on the local synteny. RT: retrotransposition. Genes are shown corresponding to the strand of GOI in order to show the homology between neighboring genes. Homology between two neighboring genes are defined by Blastp E-value < 1e-5.

Another case where local synteny clarifies the history of closely related groups of duplicates can be seen in a mouse-rat many-to-many Inparanoid ortholog group including RT copies (Figure 7). This example contains two members from each species, ENSMUSG00000013701 (MGI symbol: Timm23 referred as Mm1 in the figure), ENSMUSG00000069622 (Timm23 as Mm2) and ENSRNOG00000019811 (Timm23 as Rn1), ENSRNOG00000032900 (TIM23_RAT as Rn2), where each has one known transcript on different chromosomes. Again, neither Inparanoid nor pairwise Protdist analysis could discriminate orthologs due to identical cross-species Blastp measures (for Blastp results see Additional file 5). Ensembl has a bigger ortholog group including these four genes, but no better information about which are orthologs or RT copies. However two intron bearing genes have perfect local synteny (= 6) and an ICR = 1, and two intronless copies do not have any local syntenic match to the two intron bearing genes. Therefore, we can infer that two intron bearing genes are the main orthologs and two intronless genes are RT copies of these orthologs. Furthermore, since intronless genes are not syntenic to each other, we infer that the two intronless genes are the result of two separate RT events on each species lineage. For detailed information in tabular format, see Additional file 5.

## Discussion

### FP and FN Estimates by Using LCA

The accurate determination of orthology is critically important to comparative genomics. However it has been a challenge to compare the various orthology determination methods without a reliable gold-standard orthology dataset [15]. The statistical technique of Latent Class Analysis allows estimates of false positive and false negative rates from data based on agreement and disagreement between various ortholog definitions. Because error rates estimated in this way may be affected by which methods are included in the analysis, we must consider the FP and FN rates estimated here as relative error rates. Furthermore, these error rates might not reflect rates obtained from a genome-wide implementation of these methods. There is an ongoing effort on standardizing protein datasets for benchmarking orthology determination methods [20] which would help resolve this issue.

In Figure 3, Inparanoid and RBH agree more closely than any other pair of orthology definitions. This is in accord with the fact that Inparanoid uses the reciprocal best hit to start core ortholog identification. SBH and BLASTP have zero FN rates and higher FP rates than Inparanoid and RBH due to less stringent conditions used for ortholog detection in these methods. The order of FP rates (BLASTP > SBH > RBH) is concordant with the stringency of each method. The FP rate of local synteny based orthology falls between SBH and RBH. This is reasonable considering the fact that SBH and local synteny based orthology cannot distinguish close paralogs from ortholog pairs, but local synteny can separate RT close paralog copies from orthologs.

The FN rate of local synteny based orthology is higher than those of Blastp based orthologies (Inparanoid, RBH, SBH, and BLASTP). This may be due to distant paralogs retaining flanking genes, but diverging in their coding sequences enough to be distinguishable by Blastp. RT miscalls by Inparanoid (e.g. Figure 5) are also likely to have contributed to the relative FN rate of local synteny based orthology.

Due to the fact that OrthoMCL detected the smallest number of orthologs in any sample (data not shown) the estimated FN rates from OrthoMCL are the highest in this experiment (approximately 0.3). This is opposite from the result of Chen et al. [15] where OrthoMCL and Inparanoid were shown to have lowest estimated FP and FN rates and OrthoMCL has lower FN rates than Inparanoid. The apparent disparity between these results could be explained by the fact that LCA is designed to estimate consensus FP and FN rates without any guarantee that the estimated rates are close to absolute values, and by the difference in the species sets used in the two experiments: our dataset of five mammalian genomes and the comparatively distant seven eukaryotic genomes used in Chen et al.

### Gene Order as a Measure of Conservation

Synteny information has been used to cluster synteny blocks between two related genomes in order to detect orthologous gene pairs [8,21] and to reconstruct phylogenetic trees [22]. These synteny blocks are generally used as "genomic anchors" [11] or to place gene loss/deletion events on phylogenetic trees [23,24], not as a definitive measure to distinguish close paralogs from distant paralogs. In Zheng et al. [21], one of three methods to define orthologs between human and mouse used a genomic anchor approach. They identified synteny anchors and synteny blocks [25,26] then introduced a local synteny approach for the anchor-poor regions by accepting the pairs of genes flanked by previously identified ortholog pairs. With this approach they found 11% more orthologs than by RBH alone.

Another similar approach to local synteny was used to reconstruct phylogenetic gene trees from coding sequence similarity and local gene order [22]. In the algorithm SYNERGY, Wapinski et al. measure a synteny similarity score for a pair of genes by counting the neighboring genes in syntenic blocks. However, SYNERGY has not been tested in mammalian genomes.

Finally, MSOAR [8] uses combinatorial optimization on global gene order to identify orthologs based on minimal rearrangement scenarios. However, Fu et al. point out that the global optimization might lead to false ortholog links in some scenarios. Because our local synteny exploits proximate synteny information we expect lower false positive ratios than those obtained with MSOAR.

Most importantly, when the orthology is defined by codon sequence similarity, testing any hypothesis of selective pressures on orthologous gene presents tautological challenges. Since orthology detection by local synteny is not based on the comparison of coding sequence information between candidate orthologs, testing the selective pressure between orthologs becomes a valid comparison of largely independent variables. However, gene order also degrades over evolutionary time. How well synteny will be able to effectively identify ancient orthologs remains to be seen.

### Gene Duplication Mechanisms and Orthologs

Gene duplications are a major force in genome evolution [27]. Genes are duplicated through two main duplication mechanisms; DNA-mediated and RNA-mediated [27,28]. DNA-mediated duplications can include multiple genes and associated intergenic sequences and introns. On the other hand, RNA-mediated duplication, or retrotransposition (RT), only copies coding sequences in the duplication event. Retrotransposed genes had been considered mostly "dead on arrival", but recent studies [29,30] show that there are many functional RT copies in the human and mouse genomes.

Sometimes the difference between coding sequences of parental genes and RT copies is not large enough to distinguish the RT copy from the parent. RT copies, however, do not share introns or flanking genes with the parental paralog. Therefore, local synteny and gene structure can often separate the RT copy from the original gene. However, when neither has an intron, only local synteny will distinguish parental and RT copies. Based on our random sampling, approximately 8~10% of Pfam orthologs are intronless gene pairs.

### Gene Order Helps Illuminate Gene Family Evolution

Reconstructing phylogenetic trees informs our understanding of the evolutionary history of gene families. Using tree reconciliation between a species tree and gene tree we can identify duplication and lost events on the tree. However by distinguishing two duplication mechanisms, DNA-mediated and RNA-mediated, not just identifying duplication events, we can sometimes place duplication events in an appropriate phylogenetic context. For example, as Figure 6 and Figure 7 show, when coding sequence does not distinguish paralogs, local synteny can determine whether DNA or RNA-mediated duplication occurred first. Local synteny can also help place RT duplication events before or after speciation (Figure 7). Even when the coding sequence of RT duplicates drifts apart, pre-speciation RT genes often retain local synteny. Conversely, when RT duplicates are young enough to be indistinguishable by coding sequence comparison, synteny can discriminate between pre-speciation duplications, and independent RT duplication events in parallel lineages. Finally, local synteny information can often resolve the order of iterative DNA-mediated duplication events in large gene families (see also [9]).

## Conclusions

In this paper, we investigate the concordance between coding sequence based orthology (Inparanoid) and local synteny based orthology. In the 5 mammalian genomes studied, 93% of the sampled inter-species pairs were found to be concordant between the two orthology methods, illustrating that local synteny can largely substitute for coding sequence in identifying orthologs. However, 7% of pairs were found to be discordant. Discordance is often associated with evolutionary events like retrotransposition, iterative DNA-mediated duplication, and genome rearrangement. Analysis of discordant cases between local synteny and Inparanoid shows that local synteny can differentiate between true orthologs and recent retrogenes, can split ambiguous many-to-many orthology groups into more precise one-to-one ortholog pairs, and, when employed in a genome-wide screen, might help in highlighting possible cases of non-orthologous gene displacement by retrocopied paralogs in mammalian genomes.

## Methods

### Datasets for Ortholog Definitions

Five species analyzed (human, chimp, mouse, rat and dog) were obtained from Ensembl release 48 [31]. We only used protein coding genes in the Ensembl database. For genes with multiple alternative transcripts we used the longest transcripts as the representative ones. We used Pfam families [13] to choose ortholog candidates. Since there are more than hundreds of millions possible protein pairs among five genomes in Pfam families, we sampled our datasets in the following way. First, we randomly selected 1,000 families from 3,418 Pfam families which have at least two representative proteins from different genomes. One cross-species protein pair was selected from each Pfam family in order to avoid a bias from big families. We used 10 sample datasets for the LCA experiment and one of them was used in the discordance analysis.

### Local Synteny

Local synteny is measured by homology between the neighboring genes of two genes of interest. In this study the maximum unique homology matches between two sets of six neighboring genes (three upstream and three downstream neighbors) was used. The matches do not need to be co-linear or between genes on the same strand/orientation either (see Figure 1), which allows for genome micro-rearrangements as well as gene losses and insertions in the flanking region. The homology between neighboring genes is decided by pre-calculated Blastp [11] results in the Ensembl Compara database [14]. To avoid having high local synteny due to proximate tandem array genes, we considered the tandem array genes as one neighboring gene. Within a tandem array, each gene was counted separately.

### Orthology Definitions and LCA

We used five orthology detection methods and Ensembl orthology in comparison with our local synteny based orthology. For each of the five orthology detection methods -- namely Inparanoid [1], OrthoMCL [2], SBH (single or one-way best hits), RBH (reciprocal best hits) and BLASTP (one-way Blastp hits with the threshold) -- we used the pre-computed Blastp outputs in Ensembl database as input data. Parameters and thresholds used for each method are as follows:

1) BLASTP: homology detection using E-value cutoff (= 1e-5)

2) SBH: Single-way or One-way Best Hit. 'Best-hit' is defined as the hit (or multiple hits tied) with the highest E-value (E-value cutoff = 1e-5)

3) RBH: Reciprocal Best Hit. 'Best-hit' is defined as same as SBH (E-value cutoff = 1e-5)

4) Inparanoid (v2.0): bit score cutoff = 50 bits and sequence overlap cutoff = 0.5

5) OrthoMCL (v1.4): E-value cutoff = 1e-5 and MCL inflation index = 1.5; MCL package (v02-063) was used.

The frequency table of agreements and disagreements between orthology detection methods was calculated and used for LCA. LCA was performed using the LEM package [32] with default parameters to estimate the false positive and false negative rates. We used a basic latent model to produce Figure 3 assuming independence between various orthology detection methods. However, all methods we considered are solely or partially based on protein sequences. In order to account for these dependencies, we applied another latent model with an extra latent variable. With such a model, called latent class model with random effects or a continuous factor (CFactor) model, the responses of different tests are assumed to be independent [15,33]. Although the estimated error rates from the CFactor model (Additional file 6) are less tightly distributed than ones from the basic model, the relative values are not changed significantly compared with Figure 3. The looser distribution of values in the CFactor model is likely due to a lack of convergence in these runs. For detailed description of two LCA models and the FP/FN graph by the CFactor model, see Additional file 6.

### Intron Conservation Ratio (ICR)

Gene structure similarity is measured by the intron conservation ratio (ICR) between two intron-bearing genes [34]. For genes with multiple alternative transcripts we developed a collapsed gene model that incorporates all potential exons of that gene. Resulting exon coordinates were used to obtain the protein alignments and also to align the positions of introns. ICR between two homologous genes was calculated as the ratio of the number of positionally homologous introns divided by the total number of intron positions from the protein/intron alignment, similar to the method in Rogozin et al. [34]. Introns with less than 40BP were ignored in ICR calculation.

### Case Analysis

For the discordant cases between Inparanoid, local synteny, and/or ICR based orthology (Panels b and c of Figure 4 and part of data in Figure 4d), we investigated:

1) any significant Blastp hits other than the sampled pairs (with ICR = 0 in Figure 4c or with ICR ≥ 0.5 in Figure 4b) in order to find RT miscalls by Inparanoid (e.g., Figure 5)

2) the other Inparanoid orthologs in all 5 species in order to confirm rearrangement history in those families (with ICR > 0 in Figure 4c), and

3) Inparanoid orthologous counterparts of non-Inparanoid sampled pairs in Figure 4b and Figure 4d to confirm these sampled pairs from distant paralogs.

Mouse-rat many-to-many Inparanoid ortholog groups were collected and analyzed to reconstruct their evolutionary history by considering the genomic location and intron content of their member genes. Two example ortholog groups (Figure 66 and Figure 7) were chosen due to their unambiguous evolutionary history: for most groups it is difficult to unambiguously reconstruct the full evolution due to the presence of intermingled events.

## Authors' contributions

JJ performed the programming and the data analysis. JJ, IIM and CEN participated in the design of the study and drafted the manuscript. All authors read and approved the final manuscript.

## Supplementary Material

Additional file 1 **Various numbers of neighbors**. False positive (FP) and false negative rates (FN) of local synteny measures to the Inparanoid orthologs and Ensembl orthologs, with using different number of neighbors. Click here for file

Additional file 2 **Tabular format of Figure **5. One example of RT miscall cases by Inparanoid confirmed with local synteny and ICR also shown in tabular format. Click here for file

Additional file 3 **Example of loss of local synteny case**. One example of loss of local synteny along the evolutionary distance. Click here for file

Additional file 4 **Tabular format of Figure **6. An example of many-to-many Inparanoid ortholog groups where a SD event proceeded mouse-rat speciation. Click here for file

Additional file 5 **Tabular format of Figure **7. An example of many-to-many Inparanoid ortholog groups where RT events followed the mouse-rat speciation. Click here for file

Additional file 6 **LCA with a CFactor model**. Another LCA model to account for the dependency between orthology detection methods and a corresponding FP/FN graph. Click here for file
