# Supplementary material for: Identification of mammalian orthologs using local synteny
Source: BMC Genomics. 2009 Dec 23;10:630. doi: 10.1186/1471-2164-10-630 (PMC2807883; doi:10.1186/1471-2164-10-630)
Supplement: Additional file 1 — Various numbers of neighbors. False positive (FP) and false negative rates (FN) of local synteny measures to the Inparanoid orthologs and Ensembl orthologs, with using different number of neighbors. [file 1471-2164-10-630-S1.PDF]

Additional file 1 – Various numbers of neighbors

To access the effect of the various neighbor window sizes for measuring the local synteny, we used 6, 10 and 20 neighboring genes. Since there is no a gold standard of orthology, we calculated the false positive rate (FP) and false negative rate (FN) to Inparanoid orthologs and Ensembl orthologs. The threshold of syntenic pair for each size of locality window was chosen to make the sum of FP and FN minimized (Table A). For example, more than one match for 6 neighbors is the threshold for syntenic pair, which was used in the paper. Likewise, more than 3 matches for 10 neighbors and more than 4 matches for 20 neighbors were the thresholds respectively. Between using 6 neighbors and 10 neighbors, there was only 0.6% decrease of FP and FN, and only 1% decrease from 20 neighbors to 6 ones. In the paper, we used 6 neighbors due to computation burden.

Table A. False positive (FP) and false negative rates (FN) of local synteny measures to the Inparanoid orthologs and Ensembl orthologs, with using different number of neighbors (6, 10 and 20) and different thresholds to be syntenic.

		To Inparanoid orthologs			To Ensembl orthologs		
# neighbors	Threshold	FP	FN	Sum	FP	FN	Sum
6	>0	16.6 %	3.0 %	19.6 %	16.1 %	3.9 %	20.0 %
6	**>1**	10.3 %	4.9 %	**15.2 %**	9.4 %	5.7 %	**15.1 %**
6	>2	8.0 %	9.2 %	17.2 %	7.2 %	10.1 %	17.3 %
10	>2	10.1 %	4.7 %	14.8 %	9.1 %	5.5 %	14.6 %
10	**>3**	8.7 %	5.9 %	**14.6 %**	7.7 %	6.7 %	**14.4 %**
10	>4	8.2 %	10.5 %	18.7 %	7.4 %	11.3 %	18.7 %
20	>3	10.8 %	4.0 %	14.8 %	9.8 %	4.8 %	14.6 %
20	**>4**	9.8 %	4.4 %	**14.2 %**	8.9 %	5.1 %	**14.0 %**
20	>5	9.6 %	5.2 %	14.8 %	8.6 %	6.0 %	14.6 %
