# Supplementary material for: Identification of mammalian orthologs using local synteny
Source: BMC Genomics. 2009 Dec 23;10:630. doi: 10.1186/1471-2164-10-630 (PMC2807883; doi:10.1186/1471-2164-10-630)
Supplement: Additional file 2 — Tabular format of Figure 5. One example of RT miscall cases by Inparanoid confirmed with local synteny and ICR also shown in tabular format. [file 1471-2164-10-630-S2.PDF]

Additional file 2 – Tabular format of Figure 5

One RT miscall case by Inparanoid confirmed by local synteny and ICR. Ensembl IDs: rat A gene (ENSRNOG00000016444), rat B (ENSRNOG00000014317), dog gene (ENSCAFG00000020211).

Gene1 (# introns)	Gene2 (# introns)	Inparanoid	Ensembl	#matches	ICR	Blastp Evalue	Protdist
Rat A (0)	Dog (3)	1-to-1	Ortholog	0	n/a	9.7e-74	0.029631
Rat B (2)		No	Ortholog	6	2/3	1.3e-48	0.017762
