# Supplementary material for: Identification of mammalian orthologs using local synteny
Source: BMC Genomics. 2009 Dec 23;10:630. doi: 10.1186/1471-2164-10-630 (PMC2807883; doi:10.1186/1471-2164-10-630)
Supplement: Additional file 3 — Example of loss of local synteny case. One example of loss of local synteny along the evolutionary distance. [file 1471-2164-10-630-S3.PDF]

Additional file 3 – Example of loss of local synteny case

One example of loss of local synteny along the evolutionary distance. # matches are in the upper diagonal cells and Protdist are in the lower diagonal cells. Ensembl IDs: human (ENSG00000121031), chimp (ENSPTRG00000020229), mouse (ENSMUSG00000022672), rat (ENSRNOG00000003031) and dog (ENSCAFG00000006555).

	Human gene	Chimp gene	Mouse gene	Rat gene	Dog gene
Human gene		5	3	3	1
Chimp gene	0.004694		3	3	1
Mouse gene	0.238364	0.239234		4	1
Rat gene	0.240545	0.245377	0.087974		1
Dog gene	0.183026	0.183389	0.253876	0.257948	
