# Supplementary material for: Identification of mammalian orthologs using local synteny
Source: BMC Genomics. 2009 Dec 23;10:630. doi: 10.1186/1471-2164-10-630 (PMC2807883; doi:10.1186/1471-2164-10-630)
Supplement: Additional file 4 — Tabular format of Figure 6. An example of many-to-many Inparanoid ortholog groups where a SD event proceeded mouse-rat speciation. [file 1471-2164-10-630-S4.PDF]

Additional file 4 – Tabular format of Figure 6

Tabular format of Figure 6: an example of many-to-many Inparanoid ortholog groups where a SD event proceeded mouse-rat speciation. # matches in the upper diagonal cells and ICRs in the lower diagonal cells. Genes with the same suffices (* and #) are predicted as the orthologs duplicated before mouse-rat speciation.

	# of cds introns	ENSMUSG .1175 *	ENSMUSG .19370 #	ENSMUSG .36438	ENSRNOG .4060 *	ENSRNOG .16770 #
ENSMUSG00000001175 * Calm1	6		0	1	**4**	0
ENSMUSG00000019370 # Calm3	5	5/6		0	0	**5**
ENSMUSG00000036438 Calm3	5	5/6	5/5		1	0
ENSRNOG00000004060 * Calm1	4	4/6	4/5	4/5		0
ENSRNOG00000016770 # Calm3	4	4/6	4/5	4/5	4/4	

All Blastp hits from the mouse genes to the rat genes have E-value=5.8e-76 and score=754, and all Blastp from the rat genes to the mouse genes have E-value=5.9e-76 and score=754. All the pairwise Protdist are 0.0 except for mouse Calm1 gene with Protdist=0.006722 to the other two mouse genes.
