# Supplementary material for: Identification of mammalian orthologs using local synteny
Source: BMC Genomics. 2009 Dec 23;10:630. doi: 10.1186/1471-2164-10-630 (PMC2807883; doi:10.1186/1471-2164-10-630)
Supplement: Additional file 5 — Tabular format of Figure 7. An example of many-to-many Inparanoid ortholog groups where RT events followed the mouse-rat speciation. [file 1471-2164-10-630-S5.PDF]

Additional file 5 – Tabular format of Figure 7

Tabular format of Figure 7: an example of many-to-many Inparanoid ortholog groups where a RT event followed the mouse-rat speciation. # matches in the upper diagonal cells and Protdist in the lower diagonal cells. Genes with the suffix (*) are predicted as the orthologs and the others are independent RT copies of these orthologs.

	# of cds introns	ENSMUSG .13701 *	ENSMUSG .69622	ENSRNOG .19811 *	ENSRNOG .32900
ENSMUSG00000013701 *	6		0	**6**, **ICR=6/6**	0
ENSMUSG00000069622	0	0.0		0	0
ENSRNOG00000019811 *	6	0.018772	0.018772		0
ENSRNOG00000032900	0	0.018772	0.018463	0.0	

All Blastp results from the mouse genes to the rat genes have E-value=9.1e-110 and score=1,073, and all Blastp results from the rat genes to the mouse genes have E-value=9.3e-110 and score=1,073.
