# Supplementary material for: Identification of mammalian orthologs using local synteny
Source: BMC Genomics. 2009 Dec 23;10:630. doi: 10.1186/1471-2164-10-630 (PMC2807883; doi:10.1186/1471-2164-10-630)
Supplement: Additional file 6 — LCA with a CFactor model. Another LCA model to account for the dependency between orthology detection methods and a corresponding FP/FN graph. [file 1471-2164-10-630-S6.PDF]

Additional file 6 – LCA with a CFactor model

In order to account for the dependencies between orthology detection methods, we added an extra latent variable to the basic model (Figure A1). In such model, called latent class models with random effects or a continuous factor (a CFactor model, Figure A2), the responses of different orthology detection methods are assumed to be independent. However, since the CFactor model has more latent class and the cluster dependency from this extra variable to the predictions, there was a convergence issue. We discarded the LCA runs with poorly converged error rates, which stopped at the local optimums.

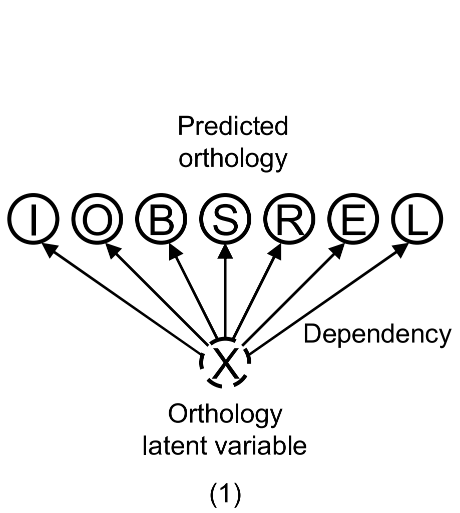

Figure A. Latent class models. (1) A basic model with one latent variable to estimate the error rates of orthology detection methods used for Figure 3. (2) A CFactor model with another a continuous factor to account for the dependencies between orthology detection methods. I: Inparanoid, O: OrthoMCL, B: BLASTP, S: SBH, R: RBH, E: Ensembl, and L: Local synteny. For the details of each method, refer to Method section.

Figure B shows the estimated error rates by using the CFactor model (Figure A2) for seven orthology detection methods. Not like Figure 3, which is from a basic model (Figure A1), the error rates of Inparanoid and RBH are overlapped each other and the estimated rates from the CFactor model are less tightly distributed, e.g. standard errors of the estimated FP and FN of Inparanoid with a basic model are 0.0008 and 0.0008 respectively, while ones from the CFactor model are 0.0013 and 0.0016. However, the relative positions of the estimated error rates from two models are very similar. In fact, the averaged distances between the error rates of Inparanoid and synteny-based method are very close; 0.0934 from a basic model and 0.0927 from the CFactor model.

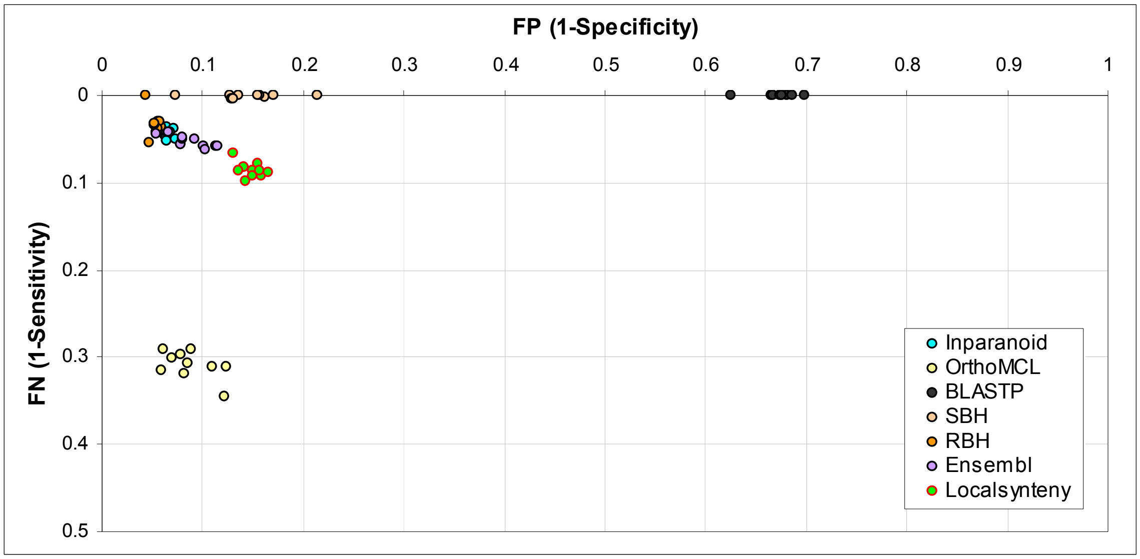

Figure B. Estimated false positive (FP) and false negative rates (FN) for seven orthology detection methods with CFactor latent model. The experiment is identical with the one explained the main text (Figure 3), except for using CFactor latent model for this figure.
